# Uncultured Amniocytes Enable Rapid and Clinically Informative Prenatal RNA Sequencing for Genetic Diagnosis

**DOI:** 10.3390/ijms27146465

**Published:** 2026-07-21

**Authors:** Jianqin Lu, Qingqing Ge, Huanyi Chen, Xinyi Zhao, Xiang Zhou, Ruibin Huang, Hang Zhou, Qiuxia Yu, Simin Yuan, Manqiu Yang, Ru Li, Dongzhi Li, Fang Fu, Can Liao

**Affiliations:** Prenatal Diagnostic Center, Guangzhou Women and Children’s Medical Center, Guangzhou Medical University, Guangzhou 510620, China; lujq7@alumni.sysu.edu.cn (J.L.);

**Keywords:** RNA sequencing, transcriptome, prenatal diagnosis, uncultured amniocytes, variant interpretation

## Abstract

RNA sequencing (RNA-seq) provides transcript-level evidence for interpreting variants of uncertain significance in monogenic disorders, but its prenatal application is limited by sample availability and processing time. This study evaluated uncultured amniocytes as a practical substrate for prenatal RNA-seq. We performed RNA-seq on 15 matched paired cultured and uncultured amniocyte samples and compared their transcriptomic profiles using principal component analysis, differential expression analysis, and pathway enrichment. We then analyzed 77 uncultured amniocyte samples collected between 16^+6^ and 29^+6^ weeks of gestation to disease-related gene expression coverage and gestational-age-associated variation. Five selected cases were further analyzed to evaluate transcript-level findings relevant to variant interpretation. Cultured and uncultured amniocytes showed distinct transcriptomic profiles, with 11,234 differentially expressed genes and culture-associated transcriptional and pathway changes. Uncultured amniocytes expressed an average of 63.4% of genes across disease-associated panels, and most disease-related genes were consistently expressed across gestational stages, although stage-associated differences in gene detectability were observed. In selected cases, uncultured amniocyte RNA-seq detected aberrant expression, aberrant splicing, allelic imbalance, and expressed sequence variants. These findings support the feasibility of prenatal RNA-seq using uncultured amniocytes and further evaluation of this approach as a complementary functional assay for prenatal variant interpretation.

## 1. Introduction

Monogenic disorders, caused by mutations in single genes, have become an important focus of prenatal genetic testing [1]. Pooled cohort data indicate that the standard prenatal diagnostic workflow, including karyotyping, chromosomal microarray analysis (CMA)/copy number variation sequencing (CNV-seq), and prenatal exome sequencing (pES), achieves an overall genetic diagnostic yield of approximately 40% in fetuses with structural anomalies [2,3,4,5,6,7]. Consequently, the majority of these cases remain genetically undiagnosed after standard invasive prenatal testing. A major limitation is the interpretation of variants of uncertain significance (VUS). Of the more than two million variants cataloged in ClinVar, approximately 36% are classified as VUS, and an additional 5% have conflicting interpretations, posing challenges for expectant parents [8]. Although whole-genome sequencing captures additional variant classes, its incremental diagnostic yield over exome sequencing has often been modest (7–19%) [9,10,11,12,13].

The transcriptome, encompassing all RNA transcripts produced under specific physiological or pathological conditions, provides a functional readout of genetic and epigenetic alterations [11,14]. Through genome-wide profiling of both transcript abundance and sequence, RNA sequencing (RNA-seq) enables systematic detection of aberrant gene expression, splicing, and monoallelic expression (MAE) [15,16,17,18]. Therefore, RNA-seq shows promise for reclassifying VUS identified by DNA sequencing and uncovering candidate genes with regulatory defects [15,19,20]. Under American College of Medical Genetics and Genomics (ACMG) and the Association for Molecular Pathology (AMP) variant interpretation guidelines, RNA-based functional evidence may be assigned different strengths depending on assay performance and specimen quality [20,21].

Multiple pilot studies have reported that RNA-seq provides an additional diagnostic yield of 7–36% beyond DNA sequencing alone in rare Mendelian disorders [11,18,22,23,24,25,26,27]. Notably, a key strength of RNA-seq lies in its ability to directly reveal splicing effects of variants that are poorly predicted by computational tools, such as synonymous variants, splice-region variants (e.g., noncanonical splice-region variants near exon–intron boundaries), and variants in non-coding regions [8,11,15,28].

However, in the prenatal context, most RNA-seq studies have primarily focused on the identification of differentially expressed genes and associated downstream functional pathways in specific conditions, such as gestational diabetes, fetal growth restriction (FGR), or preterm birth, primarily based on cultured amniocytes or amniotic fluid (AF) supernatant [29,30,31,32]. A single-cell RNA-seq study revealed that cultured amniocytes mainly comprise kidney-derived epithelial cells, smooth muscle cells, and progenitor cells; placenta-derived stromal cells and syncytiotrophoblasts; and immune cells [33]. A small-scale, proof-of-concept study characterized the expression profiles of disease-associated genes in cultured amniocytes between 16 and 21^+3^ weeks of gestation and validated the bioinformatic pipeline for RNA-seq-based functional assessment of variants [34].

In clinical practice, many amniocentesis-based prenatal diagnostic procedures are performed in the second trimester, with some extending into later gestation, making rapid turnaround essential [35,36]. Amniocyte culture typically requires 10–14 days and is therefore a major source of delay; it may also alter cellular composition and gene-expression profiles. Although Lee et al. demonstrated the feasibility of RNA-seq in cultured amniocytes, prenatal tests, including quantitative fluorescent polymerase chain reaction (QF-PCR), CMA/CNV-seq and pES, are typically performed on uncultured samples. Building on prior proof-of-concept work, the present study focused specifically on uncultured amniocytes, which align more closely with routine prenatal diagnostic workflows and avoid the additional culture step. This study aimed to (i) compare gene expression and functional pathways between cultured and uncultured amniocytes; (ii) assess gene expression in uncultured amniocytes relative to clinically accessible tissues (CATs), including whole blood and cultured fibroblasts, across disease-related gene panels and throughout the study gestational window; and (iii) evaluate variant-induced transcriptional changes in molecularly characterized cases.

## 2. Results

### 2.1. Cohort Composition

**Figure 1:**
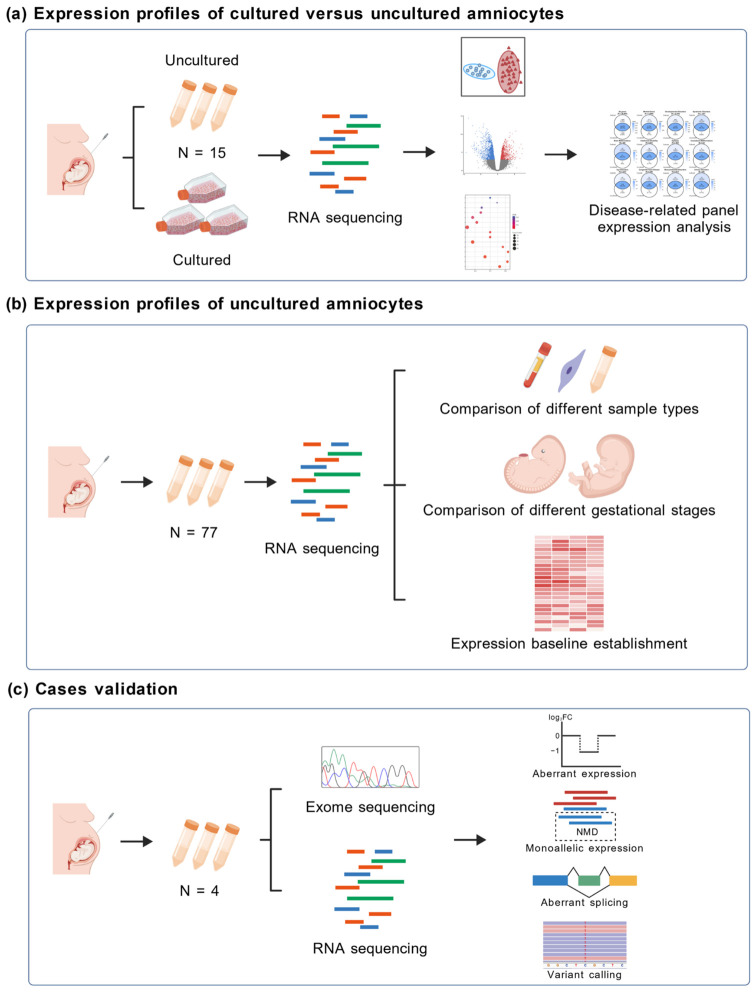
Schematic overview of the study design. To assess the feasibility of prenatal RNA-seq using uncultured amniocytes, transcriptomic profiles were first compared between uncultured and cultured amniocytes. The uncultured amniocyte cohort was then expanded for external comparison with CAT (GTEx whole blood and cultured fibroblasts) and for internal analysis across gestational stages to characterize exploratory gestational expression patterns. Finally, RNA-seq-based validation was performed in five cases carrying DNA variants expected to affect RNA expression. (Created with BioGDP.com).

The study cohort included 80 cases, which were assigned to three subgroups according to the analytical objectives (Figure 1). Subgroup 1 (n = 15) was used to compare uncultured and cultured amniocytes. Subgroup 2 (n = 77) was used for gene expression analysis of uncultured amniocytes. Subgroup 3 (n = 5) was used to evaluate uncultured amniocytes for validation of selected DNA variants. The subgroups were not mutually exclusive, as some cases contributed to more than one analysis. Comprehensive clinical characteristics and genetic findings for all cases are presented in Table S1.

### 2.2. RNA-Seq Quality Control (QC)

The final library concentration was comparable across cultured and uncultured amniocyte samples, with an overall mean of 44.5 ± 5.9 ng/µL. Sequencing yielded 44.1–123.4 million raw reads per sample, with a median of 71.7 million reads, and the mean percentage of bases with a Phred quality score ≥ 30 (Q30) was 97.3%. In total, 28,903 genes were quantified in the expression matrix, and the average duplication rate was 20.8%. Detailed quality control metrics, including mapping rate, number of detected genes, and duplication rate, are summarized in Figure S1.

### 2.3. Expression Profiles of Cultured Versus Uncultured Amniocytes

Paired uncultured and cultured amniocyte samples were collected from 15 pregnant women between 16^+6^ and 28^+4^ weeks of gestation from September 2024 to March 2025. Detailed clinical information is shown in Table S1. Except for the culture step, the two sample types were processed using the same procedures for sample handling, RNA extraction, and library preparation. The median culture time was 12 days.

**Figure 2:**
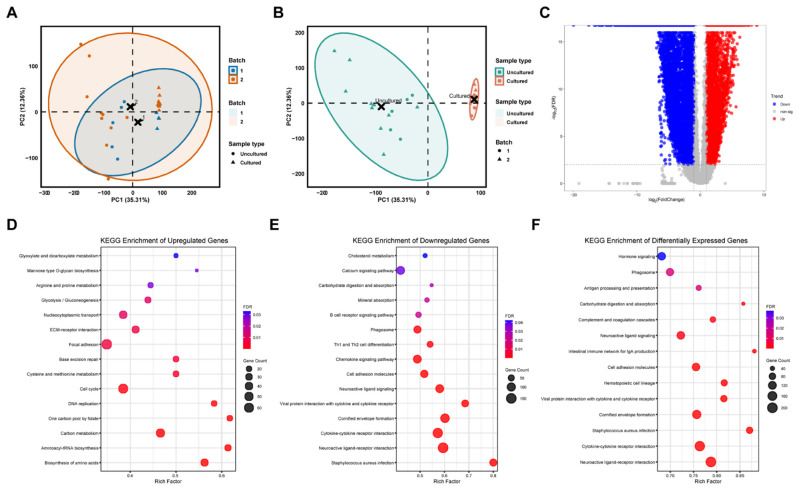
Transcriptomic differences between uncultured and cultured amniocyte samples. (**A**) PCA of paired uncultured and cultured amniocyte expression profiles (n = 15 pairs) colored by batch, showing no apparent batch-specific clustering pattern. (**B**) PCA of the same samples colored by sample type, demonstrating clear separation between uncultured and cultured amniocytes. Uncultured samples showed a broader distribution across the PCA space, indicating greater within-group variability. (**C**) Volcano plot of differentially expressed genes (DEGs) between uncultured and cultured samples. Red dots indicate significantly upregulated genes in cultured relative to uncultured amniocytes, defined by a log_2_ fold change (log_2_FC) > 1 and an FDR < 0.01, whereas blue dots indicate significantly downregulated genes, defined by a log_2_FC < −1 and an FDR < 0.01. (**D**–**F**) Bubble plots of KEGG pathway enrichment analyses of upregulated genes, downregulated genes, and all DEGs, respectively.

Principal component analysis (PCA), colored by sequencing batch, showed no clear batch-related clustering (Figure 2A). In contrast, cultured and uncultured amniocytes separated clearly when the plot was colored by sample type (Figure 2B). Uncultured samples were more widely dispersed, indicating greater inter-sample variability. Because duplication rates varied widely among uncultured samples (6.44–57.65%), we assessed whether this technical feature contributed to the observed separation. When colored by duplication-rate group, the PCA plot showed a modest duplication-related trend, mainly along PC2 (Figure S2A). Kruskal–Wallis tests showed that PC1 was significantly associated only with sample type, whereas PC2 was associated with duplication rate and sequencing batch (Figure S2B). After samples with duplication rates >20% were excluded, cultured and uncultured amniocytes remained clearly separated in the PCA plot (Figure S2C). Thus, duplication rate may contribute to a secondary component of variation but is unlikely to be the primary driver of the transcriptomic separation between the two sample types.

Paired differential expression analysis identified 11,234 differentially expressed genes between uncultured and cultured amniocytes, including 3793 genes upregulated and 7441 genes downregulated in cultured amniocytes (Figure 2C; Table S2). Kyoto Encyclopedia of Genes and Genomes (KEGG) pathway enrichment analysis identified 19 pathways significantly enriched among genes upregulated in cultured amniocytes, using a false discovery rate (FDR) threshold of 0.05. These pathways were primarily related to the cell cycle, DNA replication, and oxidative phosphorylation (Figure 2D; Table S3), consistent with adaptation to in vitro culture. Among genes downregulated in cultured amniocytes, 43 significantly enriched pathways (FDR < 0.05) were identified, including pathways associated with cell adhesion molecules, cytokine–cytokine receptor interaction, and PI3K–AKT signaling (Figure 2E; Table S4). Analysis of all differentially expressed genes identified 23 significantly enriched pathways (FDR < 0.05), primarily related to environmental information processing, organismal systems, and cellular processes (Figure 2F; Table S5).

**Figure 3:**
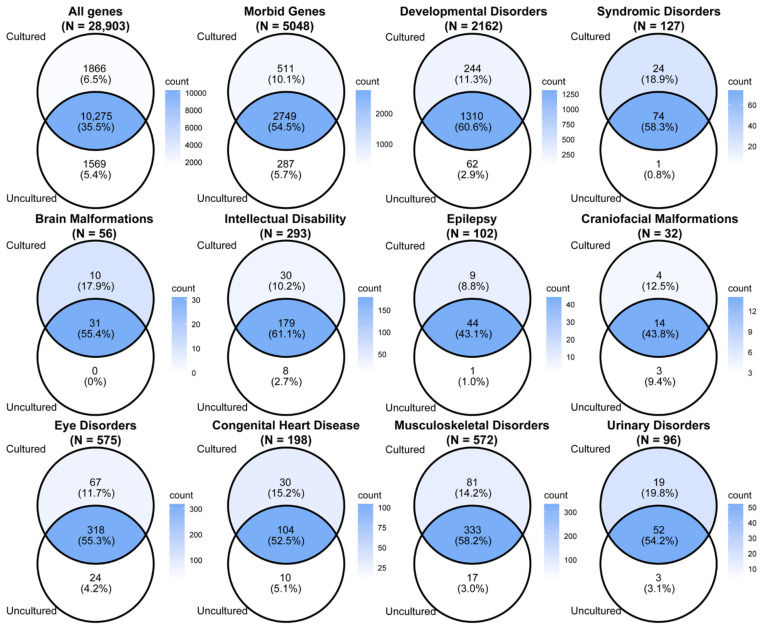
Venn diagrams showing gene expression in 12 disease-associated gene panels in cultured and uncultured amniocytes. For each panel, expressed genes (median TPM ≥ 1) were classified as shared between the two sample types or uniquely detected in either uncultured or cultured amniocytes.

We compared gene expression across 12 disease-associated gene panels between uncultured and cultured amniocytes. The genes included in each panel are listed in Table S6. For each sample type, genes with a median transcripts per million (TPM) value ≥ 1 were considered expressed. Across panels, 43.1–61.1% of genes were expressed in both groups, while a small subset showed unique expression in each sample type (Figure 3). McNemar’s test demonstrated that cultured amniocytes exhibited a significantly higher proportion of expressed genes across all panels (*p* < 0.05) except the craniofacial malformations panel (Table S7). Expression profiles of the MorbidGenes panel, which includes most genes represented in the other disease-related panels, in uncultured and cultured amniocytes are presented in Table S8. 

### 2.4. Expression Profiles of Uncultured Amniocytes

To further characterize the gene expression profile of uncultured amniocytes, we included 77 fetuses undergoing prenatal diagnostic testing for advanced maternal age (AMA) or ultrasound-detected structural anomalies. Clinical characteristics are presented in Table 1 and Table S2.

Because fetal sex may influence transcriptomic profiles, we further assessed whether sex contributed to global clustering or disease-panel expression coverage in uncultured amniocytes. PCA colored by fetal sex showed no clear separation between male and female samples, and fetal sex was not significantly associated with PC1 or PC2 scores (PC1, *p* = 0.4299; PC2, *p* = 0.112; Figure S3). Disease-panel gene expression coverage was also summarized descriptively for male and female fetuses in Table S9. These findings suggest that fetal sex was not a major driver of global transcriptomic clustering or disease-panel expression coverage in this cohort.

Given that whole blood and cultured fibroblasts are two commonly used clinically accessible tissues (CATs) in postnatal transcriptomic studies, we descriptively compared the proportions of expressed genes in GTEx whole blood and cultured fibroblasts with those in uncultured amniocytes. Among 28,903 mapped genes, 12,186 (42.2%) were expressed (median TPM ≥ 1) in uncultured amniocytes. This proportion was higher than that in whole blood (10,608 genes, 36.7%) but was slightly lower than that in cultured fibroblasts (13,001 genes, 45.0%). In the MorbidGenes panel, 3237 genes (64.1%) were expressed in uncultured amniocytes, compared with 2946 (58.4%) in whole blood and 3559 (70.5%) in cultured fibroblasts.

**Figure 4:**
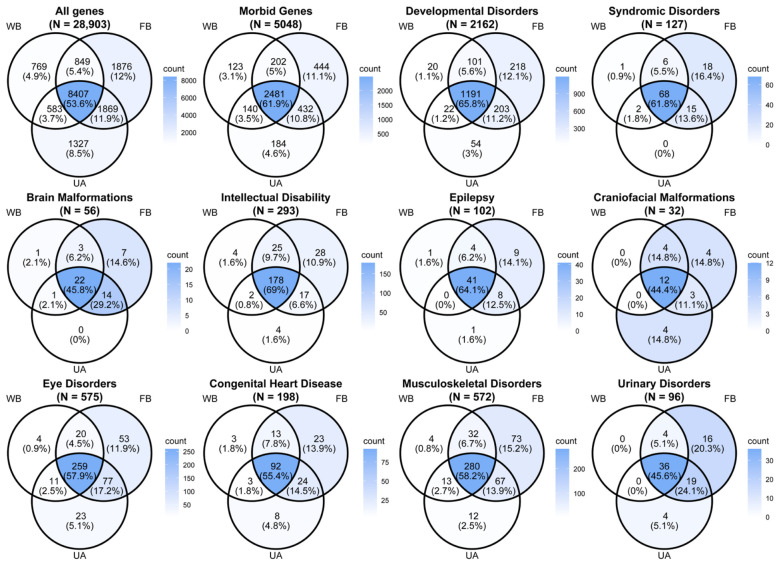
Venn diagrams showing expressed genes in 12 disease-associated gene panels across uncultured amniocytes, GTEx whole blood, and GTEx cultured fibroblasts. For each panel, genes with median TPM ≥ 1 were classified as shared among the three sample types or uniquely expressed in a specific sample type. UA, uncultured amniocytes; FB, cultured fibroblasts; WB, whole blood.

Expression overlap across the three sample types in disease-related gene panels is shown in Figure 4. Overall, cultured fibroblasts showed the broadest gene expression coverage across most panels, followed by uncultured amniocytes. Descriptively, uncultured amniocytes showed broader expression coverage than GTEx whole blood in the MorbidGenes, developmental disorder, eye disorder, musculoskeletal disorder, and urinary disorder panels (Table S10).

On average, 63.4% of genes across the disease-related panels were expressed in uncultured amniocytes. Expression coverage was highest in the intellectual disability, developmental disorder, and syndromic disorder panels. The largest proportions of genes uniquely detected in uncultured amniocytes were observed in the craniofacial malformation, urinary disorder, and congenital heart disease panels. However, these panel-level patterns are descriptive and do not establish differential clinical utility across fetal phenotypes; the informativeness of RNA-seq should be evaluated for each candidate gene. The epilepsy and craniofacial malformation panels showed the lowest overall expression coverage, at 49.0% and 59.4%, respectively.

**Figure 5:**
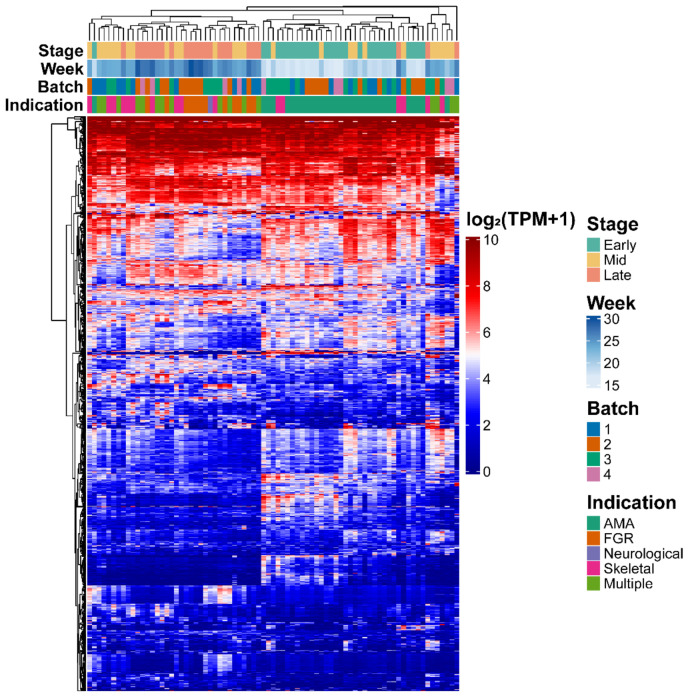
Hierarchical clustering of uncultured amniocytes across gestational ages. Hierarchical clustering (Euclidean distance) using the top 1000 most variable genes across uncultured amniocyte samples. Samples are annotated by gestational stage, gestational week, batch, and clinical indication. AMA, advanced maternal age; FGR, fetal growth restriction.

To characterize gestational-stage-associated transcriptomic patterns in uncultured amniocytes, we first performed hierarchical clustering using the top 1000 most variable genes. Hierarchical clustering showed partial gestational-stage-associated structure, with Early- and Late-stage samples exhibiting relatively distinct expression profiles (Figure 5). Mid-stage samples also showed stage-related clustering, although several overlapped with adjacent stages, suggesting gradual transcriptomic transitions across gestation.

Before further analyses, sequencing quality metrics were examined across stages. Violin plots and sample-level heatmaps showed generally comparable QC profiles across Early-, Mid-, and Late-stage samples, with no obvious stage-related imbalance in duplication rates or batch distribution (Figure S1C,D), indicating a similar distribution of technical variables across gestational stages.

**Figure 6:**
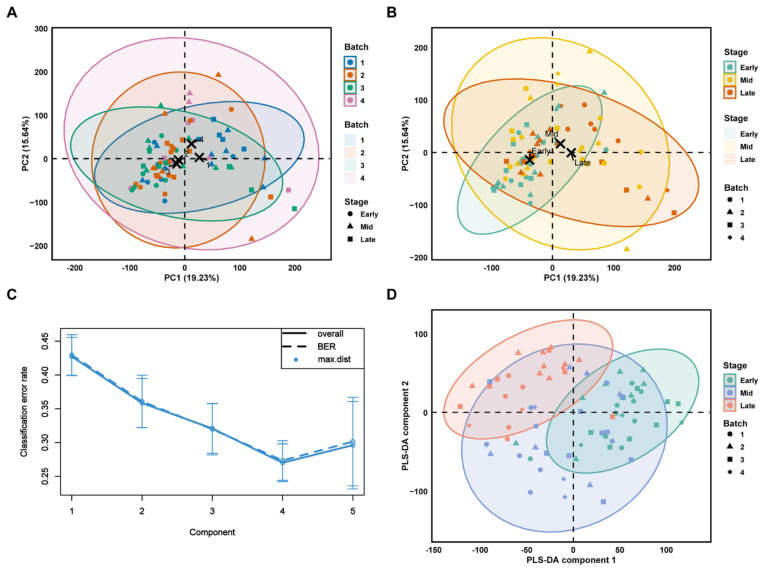
Multivariate analysis of uncultured amniocytes across gestational stages. (**A**) PCA of uncultured amniocyte expression profiles colored by sequencing batch, showing no apparent batch-specific clustering pattern across the sequencing batches. (**B**) PCA of the same samples colored by gestational stage (Early, Mid, and Late), showing limited visual separation among groups, although PC1 scores were significantly associated with gestational stage. (**C**) Optimization of the PLS-DA model. Classification error rates were evaluated across increasing numbers of latent components using threefold cross-validation. (**D**) PLS-DA score plot of Components 1 and 2. Early- and Late-stage samples exhibited relatively distinct clustering patterns, whereas Mid-stage samples showed intermediate distributions between the two stages. BER, balanced error rate; max.dist, maximum distance; PLS-DA, partial least squares discriminant analysis.

PCA of samples grouped by sequencing batch showed no apparent batch-specific clustering pattern (Figure 6A). Likewise, PCA stratified by gestational stage showed limited separation among Early-, Mid-, and Late-stage samples (Figure 6B). However, Kruskal–Wallis testing identified a significant association between gestational stage and PC1 scores (*p* = 0.001), whereas no significant association was observed for PC2, suggesting that gestational age contributes to transcriptomic variation despite the absence of clearly discrete clustering patterns. As a sensitivity analysis, PCA was repeated after adjustment for sequencing batch. The overall distribution of the samples remained similar, and the limited gestational-stage-associated pattern was retained in the batch-adjusted PCA (Figure S4). These findings indicate that the observed gestational-age-related transcriptomic signal was unlikely to be explained solely by sequencing batch.

We therefore applied partial least squares discriminant analysis (PLS-DA) as an exploratory supervised analysis. Because PLS-DA can accentuate group separation, the results were interpreted together with PCA and cross-validation findings. A four-component model was selected based on the lowest classification error rate during cross-validation (Figure 6C). PLS-DA score plots based on Components 1 and 2 showed partial stage-associated separation, with Early- and Late-stage samples showing relatively distinct distributions, whereas Mid-stage samples displayed intermediate distributions and overlapped with adjacent stages (Figure 6D). Stratified threefold cross-validation repeated 10 times showed generally stable model performance, with a mean accuracy of 0.75 ± 0.09 and a mean balanced error rate (BER) of 0.25 ± 0.09 (Figure S5A). Consistent with the score plots, the cross-validated confusion matrix indicated that Mid-stage samples were more frequently classified into adjacent stages, whereas Early- and Late-stage samples showed relatively better classification performance (Figure S5B). To assess whether the observed discrimination exceeded chance, permutation testing was performed by randomly permuting gestational-stage labels and repeating the same cross-validation procedure. The cross-validated accuracy was significantly higher and the BER significantly lower than those obtained under permuted labels (permutation *p* = 0.001 for both metrics; Figure S5C,D). Together, these findings support a gestational-age-related transcriptomic signal, while suggesting gradual transcriptomic changes across gestation rather than sharply discrete stage-specific expression profiles.

**Figure 7:**
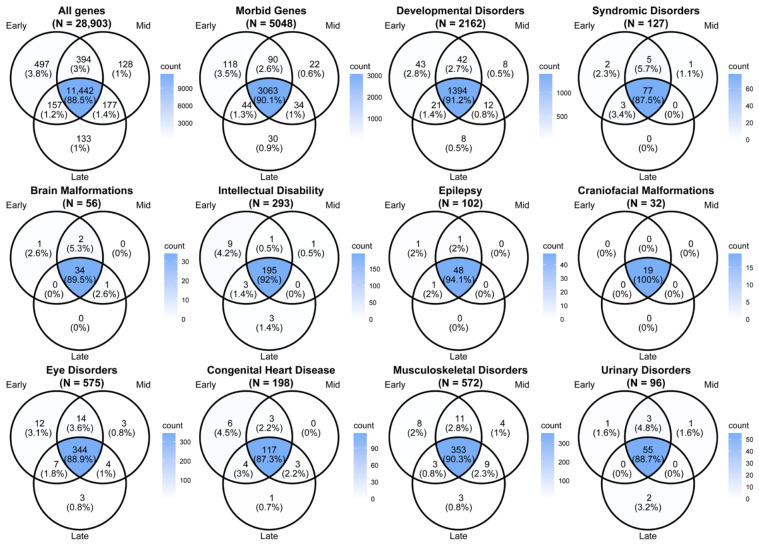
Venn diagrams showing expressed genes in 12 disease-associated gene panels of uncultured amniocytes from the Early-stage, Mid-stage and Late-stage groups. For each panel, genes with median TPM ≥ 1 were classified as shared among the three gestational stages or uniquely expressed in one stage.

Venn analysis of disease-associated gene panels across the three gestational stages identified 497, 128, and 133 uniquely expressed genes in the Early-, Mid-, and Late-stage groups, respectively, among all detectable genes (Figure 7). Within the clinically relevant MorbidGenes panel, 118 genes were detected exclusively in the Early-stage group, compared with 22 and 30 genes in the Mid- and Late-stage groups, respectively.

Overall, most expressed genes were shared across all three gestational stages, whereas only a small subset showed stage-specific expression. Descriptively, the Early-stage group showed broader expression coverage than the Mid- and Late-stage groups among all detectable genes and within several disease-associated panels. The numbers and proportions of expressed genes across the three gestational-stage groups are summarized in Table S11. These findings indicate that most clinically relevant genes were consistently detectable throughout the study gestational window, while a subset showed gestational-stage-associated expression patterns. Detailed expression profiles of MorbidGenes across gestational stages are provided in Table S12.

### 2.5. Case Validation

To illustrate the potential of transcriptome-based functional validation using uncultured amniocytes, we performed RNA-seq in cases carrying variants predicted to alter gene expression or splicing, provided that the genes of interest were expressed in uncultured amniocytes at the corresponding gestational age.

#### 2.5.1. Aberrant Expression

Case 76. At 24^+5^ weeks of gestation, ultrasonography in the fetus of a 30-year-old Chinese woman suggested tetralogy of Fallot, aberrant right subclavian artery and bilateral talipes equinovarus. Amniocentesis was performed at 26^+4^ weeks’ gestation. CMA and pES in the index pregnancy identified a de novo pathogenic 22q11.21 microdeletion spanning chr22:18,648,856–21,800,471 (GRCh37/hg19).

**Figure 8:**
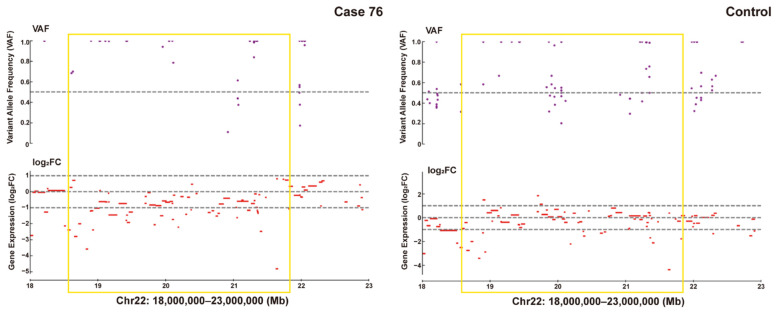
Landscape of variant allele frequencies (VAFs) and differential gene expression within the deletion region in Case 76. The yellow box indicates the 22q11.21 deletion region (18,648,856–21,800,471). Compared with control samples, Case 76 showed a higher proportion of transcribed variant sites with near-monoallelic RNA VAFs, consistent with allelic imbalance resulting from copy-number loss of the corresponding region. Most genes within the deleted region also exhibited an approximately 50% reduction in transcript abundance. FC, fold change; VAF, variant allele frequency.

Variant allele frequencies (VAFs) and gene expression fold changes (FCs) derived from RNA-seq are shown in Figure 8. VAF represents the proportion of reads supporting a specific variant at a given locus. Gene-level expression fold changes were calculated by comparing Case 76 with a reference cohort comprising samples collected at similar gestational ages. For the representative control sample, fold changes were calculated using the remaining samples with similar gestational ages as a leave-one-out reference.

In Case 76, most variants within the microdeletion region showed VAFs >0.8, indicating apparent MAE, whereas the control sample showed VAFs centered around 0.5, consistent with biallelic expression at heterozygous diploid sites. The shift toward near-monoallelic RNA VAFs in Case 76 is consistent with allelic imbalance resulting from hemizygosity after deletion of one copy of the affected region. In parallel, most genes within the deleted region showed log_2_FC values around −1, indicating an approximately 50% reduction in transcript levels consistent with a gene-dosage effect. The corresponding genes in the control sample remained near baseline expression.

**Figure 9:**
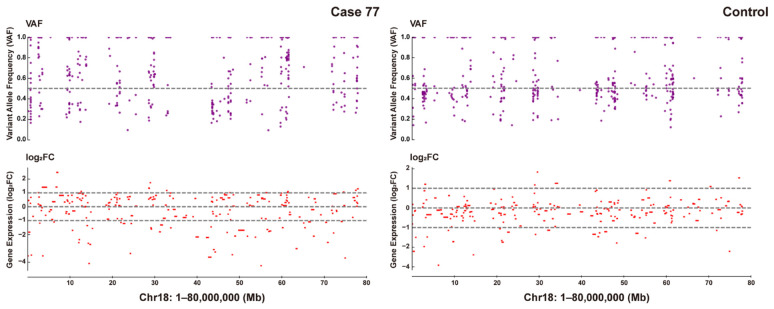
Landscape of VAFs and differential gene expression changes on chromosome 18 in Case 77. Compared with the control sample, the trisomy 18 case showed two predominant RNA VAF clusters around 0.33 and 0.67, consistent with the expected allelic dosage ratios resulting from the presence of three chromosome 18 copies. Most genes on chromosome 18 also exhibited increased transcript abundance, whereas the corresponding genes in the control sample remained near baseline expression. FC, fold change; VAF, variant allele frequency.

Case 77. At 23^+6^ weeks of gestation, ultrasonography in the fetus of a 30-year-old Chinese woman suggested agenesis of the corpus callosum, coarctation of the aorta, ventricular septal defect, left renal agenesis and right duplex kidney. Amniocentesis was performed at 25 weeks’ gestation. CMA in the index pregnancy identified a de novo pathogenic 18-trisomy. VAFs and gene expression FCs derived from RNA-seq are shown in Figure 9.

In case 77, most informative transcribed variants on chromosome 18 showed RNA VAFs clustering around 0.33 or 0.67, whereas those in the control sample were generally centered around 0.5. The two shifted VAF clusters in the trisomy 18 sample are consistent with the expected allelic dosage ratios produced by the presence of three chromosome copies. In parallel, most genes on chromosome 18 showed positive log_2_FC values, indicating increased transcript abundance, while the corresponding genes in the control sample remained near baseline expression.

**Figure 10:**
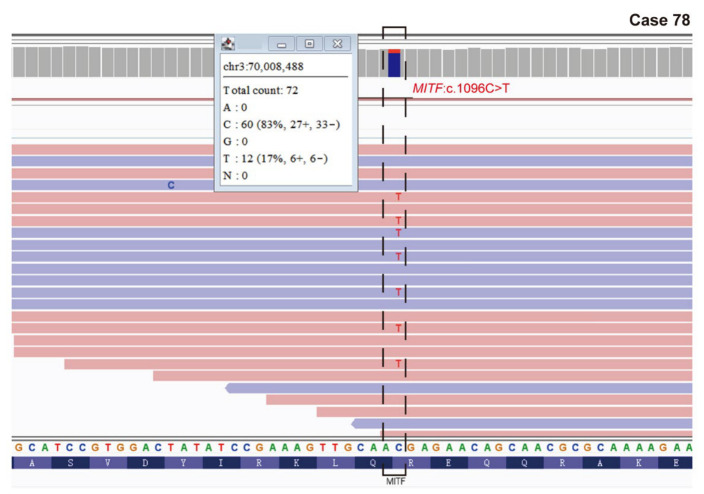
IGV profile of Case 78. RNA-seq reads showed marked allelic imbalance, with predominant expression of the reference allele (>80%), consistent with NMD induced by *MITF*:c.1096C>T (p.Arg366Ter).

Case 78. At 24^+6^ weeks of gestation, ultrasonography in the fetus of a 32-year-old Chinese woman suggested tetralogy of Fallot. Amniocentesis was performed at 25^+1^ weeks’ gestation. Prenatal trio-ES in the index pregnancy identified an incidental de novo heterozygous pathogenic nonsense variant NM_001354604.2:c.1096C>T (p.Arg366Ter) in *MITF* (MIM 156845). The Integrative Genomics Viewer (IGV) profile of RNA-seq reads from Case 78 is shown in Figure 10. Of the informative RNA-seq reads, 83.3% (60/72) mapped to the reference allele and 16.7% (12/72) supported the variant allele. Because the heterozygous nonsense variant itself was directly covered by RNA-seq reads, the underrepresented allele could be assigned to the mutant allele without additional phasing. Given that this premature termination variant was predicted to trigger nonsense-mediated mRNA decay (NMD), the marked reduction of mutant-allele reads provides transcript-level evidence supporting NMD-mediated depletion of the mutant transcript. However, as this was an incidental finding, the variant was not considered sufficient to explain the fetal ultrasound phenotype.

#### 2.5.2. Aberrant Splicing

Case 79. The fetus from a 24-year-old Chinese woman was suspected to have malformations of cortical development on ultrasonography, and amniocentesis was performed at 27^+1^ weeks’ gestation. Prenatal trio-ES in the index pregnancy identified compound heterozygous variants of uncertain significance in *SLC5A6* (MIM 604024): a paternally inherited missense variant NM_021095.4:c.457A>G (p.Met153Val) and a maternally inherited synonymous variant NM_021095.4:c.678C>T (p.Gly226=). The synonymous variant was selected for RNA-seq validation based on SpliceAI prediction of a high probability of cryptic splice-site activation. The resulting 62 bp partial deletion within exon 7 was predicted to introduce a premature termination codon, supporting its potential pathogenicity.

**Figure 11:**
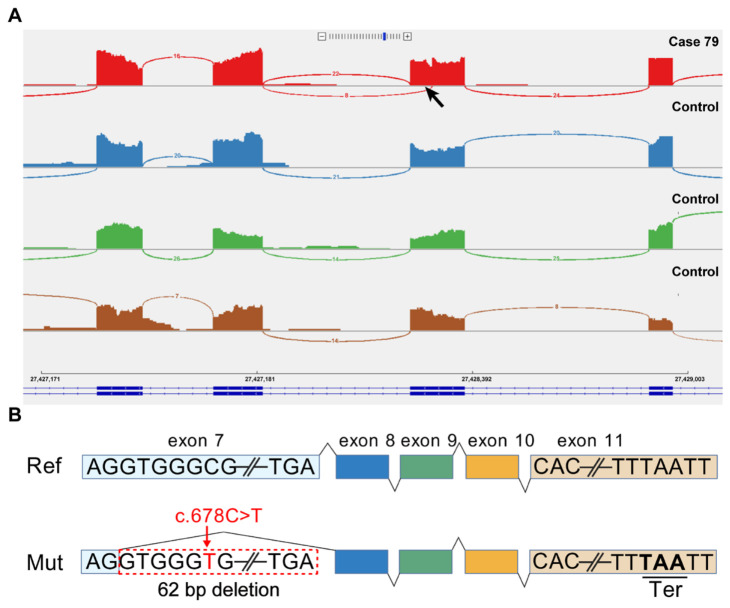
RNA sequencing demonstrates aberrant splicing caused by a synonymous variant in Case 79. (**A**) Sashimi plot showing eight reads supporting a 62 bp deletion in exon 7 (red), induced by *SLC5A6*:c.678C>T (p.Gly226=). This aberrant junction was absent in control samples (blue and green). The black arrow indicates the position of the synonymous variant. (**B**) Schematic diagram showing the frameshift caused by the exon 7 deletion and the resulting premature termination codon.

The sashimi plot of RNA-seq reads of Case 79 is shown in Figure 11A. Eight of 30 junction-spanning reads supported a 62 bp deletion in exon 7, which was absent in control samples. RNA-seq of uncultured amniocytes confirmed aberrant splicing associated with the synonymous variant at the transcriptome level (Figure 11B), leading to a revised transcript consequence of c.673_734del (p.Val225AlafsTer127). This RNA-confirmed loss-of-function effect provided evidence for reclassification of the variant from VUS to pathogenic (PVS1 + PM2_Supporting + PP1). However, because the *SLC5A6* missense variant remained a VUS (PM3 + PM2_Supporting + PP3 + PP1) and *SLC5A6*-related disease is inherited in an autosomal recessive manner, the overall molecular diagnosis in this fetus remained inconclusive.

#### 2.5.3. Variant Calling

**Figure 12:**
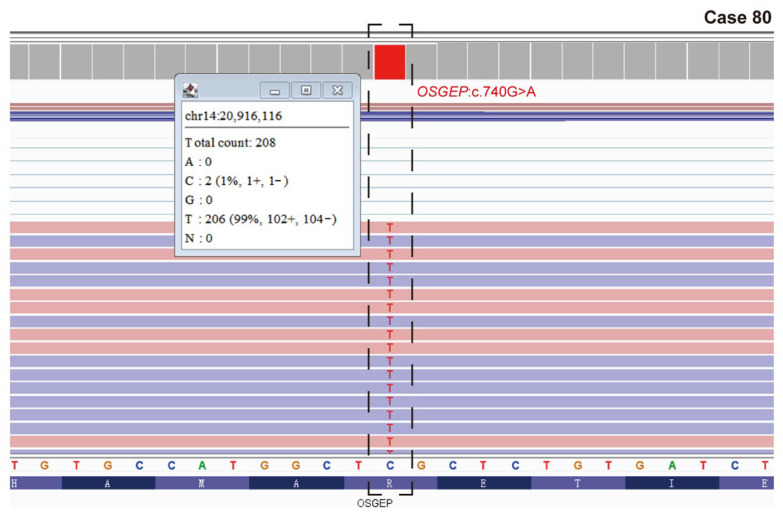
IGV profile of Case 80. RNA-seq reads showed that 99.0% of informative reads (206/208) supported the homozygous missense variant *OSGEP*:c.740G>A (p.Arg247Gln).

Case 80. At 23^+6^ weeks’ gestation, ultrasonography in the fetus of a 39-year-old Chinese woman suggested FGR. Amniocentesis was performed at 24 weeks’ gestation. Prenatal trio-ES in the index pregnancy identified a homozygous pathogenic missense variant, NM_017807.4:c.740G>A (p.Arg247Gln), in *OSGEP* (MIM 610107). The IGV profile of RNA-seq reads of Case 80 is shown in Figure 12, with 99.0% (206/208) of informative reads supporting the variant allele. These findings show that RNA-seq can provide complementary transcript-level support for the DNA-based finding by demonstrating that the same variant is detectable in the corresponding transcript.

## 3. Discussion

RNA-seq has been increasingly used in postnatal rare disease studies, where it can provide additional diagnostic information for cases that remain unresolved after DNA sequencing [11,18,22,23,24,25,26,37]. In the prenatal setting, transcript-level evidence may be particularly useful because variant interpretation often needs to be completed within a limited clinical timeframe. However, the choice of sample is more constrained before birth. Amniocytes are the most commonly obtained substrate during invasive prenatal diagnosis. Although RNA-seq has previously been shown to be feasible using cultured amniocytes [34], most prenatal genetic tests other than karyotyping are performed on uncultured amniocytes. This raises a practical question of whether uncultured amniocytes can be used directly for prenatal RNA-seq.

This study extends prior proof-of-concept work using cultured amniocytes by systematically evaluating uncultured amniocytes in the prenatal RNA-seq setting. The main contribution of our study is therefore not to establish the feasibility of amniocyte RNA-seq in general, but to assess whether the culture step can be bypassed while retaining clinically relevant transcript-level information. To this end, we compared paired cultured and uncultured amniocytes and further characterized uncultured amniocytes across 16^+6^–29^+6^ weeks of gestation. Our results indicate that uncultured amniocytes can serve as a practical substrate for RNA-seq and can provide information on aberrant expression, splicing changes, allelic imbalance, and expressed sequence variants.

Consistent with previous reports that in vitro culture can influence the detectable transcriptome [38,39], the paired analysis showed clear transcriptomic differences between cultured and uncultured amniocytes in our study. PCA separated the two sample types, and differential expression analysis identified a large number of differentially expressed genes. These differences are likely to reflect both culture-induced transcriptional remodeling and culture-associated changes in cellular composition. The enrichment of cell cycle, DNA replication, and oxidative phosphorylation pathways among genes upregulated in cultured amniocytes is consistent with proliferative adaptation during in vitro culture. In addition, uncultured amniocytes represent a heterogeneous mixture of fetal and extra-fetal cell populations [40], and culture may selectively expand cells with greater proliferative capacity, resulting in a more uniform transcriptional profile [41]. Thus, the greater inter-sample variability observed in uncultured samples may partly reflect the cellular heterogeneity, whereas cultured samples may represent a selected population adapted to in vitro growth. The relatively broad gestational age range of the paired samples may also have contributed to variability among uncultured samples [42]. Because several uncultured samples had high duplication rates, sensitivity analysis excluding highly duplicated samples indicated that the main difference between the two sample types was unlikely to be explained by duplication rate alone. However, because this study used bulk RNA-seq, we cannot quantitatively distinguish transcriptional changes within the same cell types from shifts in cell-type proportions.

These results also show that the two sample types have different strengths. Cultured amniocytes offer higher RNA yield and broader gene detection, which can be advantageous for transcriptome analysis. However, culture requires additional time and may alter the original cellular composition and expression profile. Uncultured amniocytes avoid this delay and are more consistent with the workflow already used for QF-PCR, CMA/CNV-seq, and pES. Although they may have lower RNA input and somewhat reduced detection sensitivity, they provide a pre-culture transcriptomic profile and may reduce biases introduced by culture-related transcriptional changes or cellular selection.

We also descriptively compared uncultured amniocytes with GTEx whole blood and cultured fibroblasts, two tissue types commonly used in postnatal RNA-seq studies. This comparison was intended to provide a broad reference for gene detectability rather than to infer the relative diagnostic suitability of different tissues. Uncultured amniocytes showed disease-related gene expression coverage broadly comparable to these postnatal tissues, suggesting that they may be a feasible prenatal substrate when the candidate gene is sufficiently expressed. Notably, these comparisons are confounded by differences in developmental stage, tissue origin, sample processing, sequencing platform, and data source.

The different expression rates across disorder panels may reflect both biological and panel composition factors. Human gene expression is highly tissue- and cell-type-specific, and RNA-seq-based interpretation depends on whether the gene of interest is sufficiently expressed in the sampled tissue [23,43]. Uncultured amniocytes represent a heterogeneous mixture of fetal and extra-fetal cells rather than a single disease-target tissue, and therefore their expression coverage is expected to vary according to the tissue specificity and developmental timing of the genes included in each disorder panel [33,34]. Genes included in developmental disorder and syndromic disorder panels may be enriched for broadly expressed regulatory genes, including developmental regulators, chromatin modifiers, transcriptional regulators, and signaling pathway genes [44,45,46]. Therefore, the clinical utility of uncultured amniocyte RNA-seq should be interpreted at the individual gene level rather than solely at the disorder-panel level.

Gestational age is another factor that needs to be considered when interpreting uncultured amniocyte RNA-seq data. In our exploratory analysis, most disease-related genes were expressed consistently across the Early-, Mid-, and Late-stage groups, but gestational age-related transcriptomic variation was still detectable. Unsupervised analysis showed an association between gestational stage and transcriptomic variation, and supervised analysis showed partial stage-associated separation. Because PLS-DA is a supervised method and may accentuate group separation, these results should be interpreted cautiously and together with cross-validation and permutation testing. The observed PLS-DA performance exceeded that expected under permuted labels, supporting a gestational-age-related transcriptomic signal rather than random group separation. However, Mid-stage samples were more likely to overlap with adjacent stages, which is compatible with gradual transcriptomic changes across gestation rather than sharply separated stage-specific profiles. These gestational window groups were defined primarily for exploratory analysis based on the available sample distribution, and therefore should not be interpreted as discrete biological stages. These observations suggest that gestational age should be considered when building reference sets and evaluating candidate genes, especially for genes with developmental-stage-dependent expression.

The case analyses further illustrate how uncultured amniocyte RNA-seq may contribute to prenatal variant interpretation. RNA-seq is particularly informative for variants expected to alter transcript abundance or splicing, including nonsense variants, splice-region variants, synonymous variants with predicted splice effects, and regulatory or non-coding variants [10,18,47,48]. Current variant interpretation often relies on predicted protein consequences and canonical splice-site effects [8,21], but variants outside these categories may still have important RNA-level consequences [8,10,49]. In silico splicing prediction is helpful, but its reliability decreases for non-canonical splice regions and more complex splicing events [10,19,50,51,52,53]. Direct RNA evidence can therefore be valuable. In our selected cases, uncultured amniocyte RNA-seq detected reduced expression within a microdeletion region, chromosome 18 gene-expression changes in a trisomy 18 case, allelic imbalance consistent with nonsense-mediated decay, aberrant splicing caused by a synonymous variant, and expression of a pathogenic sequence variant. Notably, most genes on chromosome 18 were upregulated, consistent with a gene-dosage effect, whereas a subset showed unchanged or decreased expression, indicating a complex and nonuniform transcriptional response [54,55]. These examples illustrate that RNA-seq can provide transcript-level evidence across different variant classes when the gene of interest is expressed, but they do not constitute systematic evidence of diagnostic improvement. Larger prospective studies are needed to determine its diagnostic yield and clinical utility in prenatal diagnosis.

Therefore, it is important to distinguish feasibility from clinical implementation. In this study, feasibility means that RNA-seq data can be generated from uncultured amniocytes, disease-gene expression coverage can be assessed, and transcript-level evidence can be obtained in selected cases. Clinical implementation, however, requires additional validation. This includes prospective studies to determine diagnostic yield and clinical utility, as well as gestational-age-aware reference datasets, validated interpretation thresholds, standardized laboratory and bioinformatic workflows, cost-effective turnaround times, and integration with prenatal genetic counseling.

Several caveats should be considered when applying uncultured amniocyte RNA-seq for prenatal variant interpretation. First, RNA-seq is inherently affected by tissue specificity. A disease gene may not be expressed or may be expressed at a low level in amniocytes, limiting the ability to assess its transcript-level consequence. Conversely, an RNA abnormality detected in amniocytes may not fully represent the disease-relevant tissue. Nevertheless, strong loss-of-function effects, such as nonsense-mediated decay or major splice disruption, may be detectable across multiple tissues in some settings [56,57]. Second, amniotic fluid contains a mixture of fetal cell types. This heterogeneity may broaden the range of detectable transcripts, but it may also dilute signals from the most relevant cell population. Third, short-read RNA-seq has technical limitations, including uneven coverage, possible 3′ bias, and limited ability to resolve complex isoforms, repetitive regions, or highly homologous genes [10,14,18,56]. RNA-based variant calling may provide orthogonal support for variants detected by DNA sequencing, but it should not be used as a standalone diagnostic approach because detection depends strongly on gene expression and read coverage [15,19,58]. These considerations support further evaluation of uncultured amniocyte RNA-seq as a complementary test, particularly when a candidate variant is suspected to affect transcript abundance or splicing.

This study has several limitations. The sample size was modest, and the gestational age range, although clinically relevant, was not sufficient to establish a week-specific reference baseline. In addition, because amniocentesis is invasive, the reference cohort was necessarily derived from clinically referred pregnancies, mainly because of fetal abnormalities or advanced maternal age, rather than from an unselected population with uncomplicated pregnancies. Accordingly, this cohort should be regarded as a clinically relevant prenatal reference cohort rather than a definitive normal pregnancy reference population, and referral-related factors may have contributed additional biological variability. Whether the same expression distributions apply to uncomplicated pregnancies remains to be validated in larger and more representative cohorts. Comparisons with GTEx tissues should also be interpreted with caution because the data were generated from different sources and may be affected by batch effects or platform-related differences. The use of GRCh37/hg19 also limits direct integration with newer GRCh38-based resources, although this reference build was selected to maintain consistency with our clinical prenatal sequencing workflow. Finally, because the cohort did not include samples collected beyond 29^+6^ weeks of gestation, the applicability and performance of uncultured amniocyte RNA-seq in later stages of the third trimester remain to be evaluated. These limitations should be addressed before broader clinical implementation.

## 4. Materials and Methods

### 4.1. Study Design

The sample size was calculated based on sequencing depth, coefficient of variation, magnitude of differential expression, false positive rate, and statistical power [30]. Assuming an average of 70 million reads per sample, a within-group coefficient of variation of 0.4, a twofold effect size, a type I error rate of 0.05, and 80% power, at least six subjects per group were required.

This prospective single-center study was conducted at a tertiary medical center and enrolled fetuses undergoing prenatal diagnostic testing for advanced maternal age (AMA) or ultrasound-detected structural anomalies. In AMA cases, QF-PCR and CMA/CNV-seq were offered, with karyotyping and pES additionally performed when clinically indicated. For cases with ultrasound anomalies, sequential genetic testing consisting of QF-PCR, karyotyping, CMA/CNV-seq, and pES was offered.

Because gestational age may influence fetal transcriptomic profiles and the sample size was insufficient for week-by-week comparisons, samples were stratified into three exploratory gestational window groups according to the distribution of available samples: 16^+6^–19^+6^ weeks (here termed the Early-stage group), 20^+0^–24^+6^ weeks (Mid-stage group), and 25^+0^–29^+6^ weeks (Late-stage group). These groups were used to explore broad gestational trends within the study cohort and should not be interpreted as established biological stages or trimester-based categories.

This study was approved by the Ethics Committee of Guangzhou Women and Children’s Medical Center (No.2021-356B01). Informed consent was obtained from all individual participants included in the study.

### 4.2. Sample Collection and Processing

A total of 15 mL of AF was additionally collected during amniocentesis and stored at 4 °C for no more than 4 h prior to processing.

For the uncultured group, 10 mL of AF sample was centrifuged at 300× *g* for 10 min at 4 °C. The amniocyte pellet was resuspended and centrifuged at 300× *g* for 5 min to remove residual supernatant, then preserved in 500 μL RNAlater (Thermo Fisher Scientific, Waltham, MA, USA) and immediately stored in RNase-free microcentrifuge tubes (Axygen Scientific, Union City, CA, USA) at −80 °C.

For the cultured group, 5 mL of AF sample was used for amniocyte culture in 25 cm^2^ culture flasks until approximately 70% confluence. Trypsinized amniocytes were then harvested and processed following the same centrifugation and preservation protocols established for uncultured AF samples. Because this study used a low-input RNA-seq workflow, sample input was defined by fixed AF volume rather than by a predefined number of starting amniocytes.

### 4.3. RNA Isolation and Sequencing

Frozen amniocyte pellets preserved in RNAlater were centrifuged at 3000× *g* for 5 min, and total RNA was extracted using the Ultrapure RNA Kit (Cowin Biotech, Jiangsu, China) according to the manufacturer’s instructions.

RNA quantity and quality were assessed using the Qsep400 Bio-fragment Analyzer (Houze Biotech, Zhejiang, China). Only high-quality, intact RNA samples showing clear 18S and 28S ribosomal RNA bands were selected for library preparation. For cultured AF samples, 30 ng of total RNA was used for reverse transcription. For uncultured AF samples, RNA yield was often extremely low; when accurate RNA quantification was not feasible or when the total RNA amount was <30 ng, the maximum allowable input volume of 10.5 µL RNA eluate was used for reverse transcription.

Poly(A)+ RNA was captured and reverse-transcribed into cDNA, followed by amplification using the SMART-Seq HT Kit (Takara Bio Inc., Kusatsu, Japan) according to the manufacturer’s protocol. Sequencing libraries were prepared using the VAHTS Universal Plus DNA Library Prep Kit for MGI V2 (Vazyme Biotech, Nanjing, China) and validated using the Qsep400 Bio-fragment Analyzer (Houze Biotech, Hangzhou, China). High-quality RNA libraries were sequenced on the DNBSEQ-T7 platform (MGI Tech, Shenzhen, China) with 150 bp paired-end reads. The average sequencing depth exceeded 70 million clean reads per sample (approximately 10 Gb raw data).

### 4.4. Sequencing Read Mapping and Gene Expression Analysis

Raw RNA-seq data (FASTQ files) were filtered using Trimmomatic (v0.36) [59]. Clean reads were aligned to the reference genome GRCh37/hg19 with HISAT2 (v2.2.1) [60]. This assembly was used because it was the reference genome adopted in our established in-house prenatal exome sequencing, CNV-seq, and variant annotation workflow during the study period. Maintaining the same genome build enabled direct comparison of RNA-seq findings with clinically generated DNA-based results, including sequence variants and CNV coordinates, and avoided potential ambiguity introduced by coordinate conversion. Alignments were output as BAM files, sorted with SAMtools v1.7 [61], and processed with Picard MarkDuplicates v2.23.3 (http://picard.sourceforge.net/index.shtml (accessed on 10 August 2025) to mark duplicate reads. Variants were called using the GATK v4.1.8 best-practices pipeline, generating VCF files [58]. Gene expression was quantified with RSEM v1.3.0 [62]. TPM values were used for gene detectability analyses because TPM normalizes read abundance for both gene length and sequencing depth. Genes with a median TPM ≥1 were considered expressed, whereas those with a median TPM <1 were considered not expressed. This threshold was selected a priori because TPM ≥1 is commonly used as a pragmatic cutoff to distinguish detectable expression from low-level background transcription in RNA-seq studies. Similar thresholds have been used in transcriptomic analyses [24,26,63], including amniotic fluid cell RNA-seq [34]. The same threshold was applied consistently across disease-related gene panels and gestational window groups.

### 4.5. Clustering Analysis and Differential Expression Analysis

The RSEM-derived expression matrix was used for unsupervised principal component analysis (PCA) and supervised partial least squares discriminant analysis (PLS-DA) to reduce dimensionality and assess clustering of predefined sample groups. Expression matrices used for PCA, hierarchical clustering, and PLS-DA were based on log_2_(TPM + 1) values. Potential technical and biological sources of variation were assessed using PCA visualization and statistical testing. PCA plots were colored by sequencing batch, gestational stage, duplication-rate group, or fetal sex, as appropriate. Associations of PC1 and PC2 scores with sequencing batch, gestational stage, duplication rate, and fetal sex were evaluated using Kruskal–Wallis tests. As a sensitivity analysis, batch-adjusted expression matrices were generated from log_2_(TPM + 1) values using the removeBatchEffect function in the limma package, with sequencing batch specified as the batch factor. Gestational stage was included in the design matrix to preserve stage-associated biological variation during batch-effect removal. Batch-adjusted values were used only for PCA-based sensitivity analyses and were not used to define gene expression status, which was based on the original TPM values.

Euclidean distance-based hierarchical clustering was performed on uncultured amniocytes across gestational ages using the top 1000 most variable genes to visualize inter-sample relationships. PLS-DA was used as an exploratory supervised analysis to assess gestational-stage-associated transcriptomic patterns. The optimal number of PLS-DA components was selected by threefold cross-validation. Model performance was further evaluated using stratified threefold cross-validation repeated 10 times based on held-out predictions. Overall accuracy, balanced error rate, and the cross-validated confusion matrix were used to evaluate the discriminative pattern among the three gestational stage groups. To assess whether the observed discrimination exceeded chance, permutation testing was performed by randomly permuting gestational-stage labels 1000 times and repeating the same cross-validation procedure. The observed accuracy and BER were then compared with the corresponding null distributions generated from permuted labels. All analyses were conducted in R (v4.5.1), using the stats package for PCA, the mixOmics and ropls packages for PLS-DA, and the ComplexHeatmap package for clustering visualization.

EBSeq (v1.26.0) was used to identify differentially expressed genes (DEGs) between predefined sample groups [64]. For the comparison between cultured and uncultured amniocytes, differential expression analysis was performed using a paired design. Each cultured sample was matched to the corresponding uncultured sample obtained from the same pregnancy. To detect expression outliers in individual cases, a reference cohort was constructed from other samples within the same gestational window, under the assumption of genetic heterogeneity [22,26]. DEGs were defined by a false discovery rate (FDR) <0.01 and an absolute log_2_ fold change >1 (|log_2_FC| > 1). KEGG pathway enrichment analysis of DEGs was performed using the clusterProfiler package in R (v4.5.1).

### 4.6. Gene Lists for Evaluating the Expression of Clinically Relevant Genes

To evaluate the expression of clinically relevant genes in uncultured amniocytes, we integrated gene panels of interest from the Gene2Phenotype Projects (https://www.ebi.ac.uk/gene2phenotype (accessed on 16 September 2025)), ClinGen Gene Curation Expert Panels (https://search.clinicalgenome.org/kb/affiliate?page=1&size=25&search= (accessed on 16 September 2025)) and relevant literature [65,66]. Only genes with Moderate, Strong, or Definitive gene–disease validity were included. The MorbidGenes panel provides a comprehensive overview of diagnostically relevant rare disease genes based on multiple public databases, including OMIM, PanelApp, SysNDD, ClinVar, HGMD and GenCC [67]. Details of the genes included in each panel are provided in Table S1.

### 4.7. Statistical Analysis

Statistical analyses were performed in R (v4.5.1). Normally distributed continuous variables are presented as mean ± standard deviation (SD), whereas non-normally distributed variables are presented as median and interquartile range (IQR). Categorical variables are presented as percentages. Paired comparisons of the proportions of expressed genes between cultured and uncultured amniocytes were performed using McNemar’s test or exact McNemar’s test, as appropriate, and *p* values were adjusted for multiple comparisons using the Benjamini–Hochberg method.

## 5. Conclusions

This study demonstrates the feasibility of using uncultured amniocytes as a practical substrate for prenatal RNA sequencing. By bypassing culture, uncultured samples provide a pre-culture transcriptomic profile and avoid culture-associated transcriptional changes. They showed broad but variable expression coverage across disease-related gene panels, emphasizing the need to assess expression at the individual gene level before interpretation. Gestational-age-related transcriptomic variation was detectable and should be considered when constructing reference datasets and interpreting RNA-seq findings. In selected cases, uncultured amniocyte RNA-seq complemented prenatal variant interpretation by providing evidence of aberrant expression, aberrant splicing, allelic imbalance, and expressed sequence variants. Larger prospective studies are required to determine diagnostic yield and clinical utility and to establish reference datasets, standardized workflows, and validated interpretation criteria.

## Figures and Tables

**Table 1:** Clinical characteristics of the 77 uncultured amniocyte samples.

Characteristic	Value
Maternal age (years)	34.7 (29.7, 39.1)
Gestational age at invasive testing (weeks)	23 + 3 (18 + 1, 25 + 4)
Fetal sex	
Male	46 (59.7%)
Female	31 (40.3%)
Primary phenotype	
Advanced maternal age	32 (41.6%)
FGR	12 (15.6%)
Neurological abnormalities	1 (1.3%)
Musculoskeletal abnormalities	16 (20.8%)
Multiple structural abnormalities	16 (20.8%)
Genetic testing	
CMA/CNV-seq	33 (42.9%)
CMA/CNV-seq + ES	44 (57.1%)
Genetic outcomes	
Diagnostic	7 (9.1%)
Small variant	3 (3.9%)
CNV	4 (5.2%)
Incidental finding	5 (6.5%)
Small variant	4 (5.2%)
CNV	1 (1.3%)
VUS	6 (7.8%)
Small variant	3 (3.9%)
CNV	3 (3.9%)
Non-diagnostic	59 (76.6%)

Continuous variables are presented as median (IQR), and categorical variables as n (%). CMA, chromosomal microarray analysis; CNV, copy number variant; CNV-seq, copy number variation sequencing; ES, exome sequencing; FGR, fetal growth restriction; VUS, variant of uncertain significance.

## Data Availability

The raw sequencing data supporting the conclusions of this article will be made available by the authors, without undue reservation, upon reasonable request.

## Supplementary Materials

The following supporting information can be downloaded at: https://www.mdpi.com/article/10.3390/ijms27146465/s1.

## Abbreviations

The following abbreviations are used in this manuscript:

ACMG	American College of Medical Genetics and Genomics
AF	Amniotic fluid
AMA	Advanced maternal age
AMP	Association for Molecular Pathology
CAT	Clinically accessible tissues
CMA	Chromosomal microarray analysis
CNV-seq	Copy number variation sequencing
DEGs	Differentially expressed genes
FC	Fold change
FDR	False discovery rate
FGR	Fetal growth restriction
IGV	Integrative Genomics Viewer
IQR	Interquartile range
MAE	Monoallelic expression
NMD	Nonsense-mediated mRNA decay
PCA	Principal component analysis
pES	Prenatal exome sequencing
PLS-DA	Partial least squares discriminant analysis
QC	Quality control
QF-PCR	Quantitative fluorescent polymerase chain reaction
RNA-seq	RNA sequencing
SD	Standard deviation
TPM	Transcripts per million
VAFs	Variant allele frequencies
VUS	Variants of uncertain significance
