# Supplementary material for: Uncultured Amniocytes Enable Rapid and Clinically Informative Prenatal RNA Sequencing for Genetic Diagnosis
Source: Int J Mol Sci. 2026 Jul 21;27(14):6465. doi: 10.3390/ijms27146465 (PMC13411854; doi:10.3390/ijms27146465)
Supplement: Supplementary file 1 [file ijms-27-06465-s001.zip › Supplementary Figures.pdf]

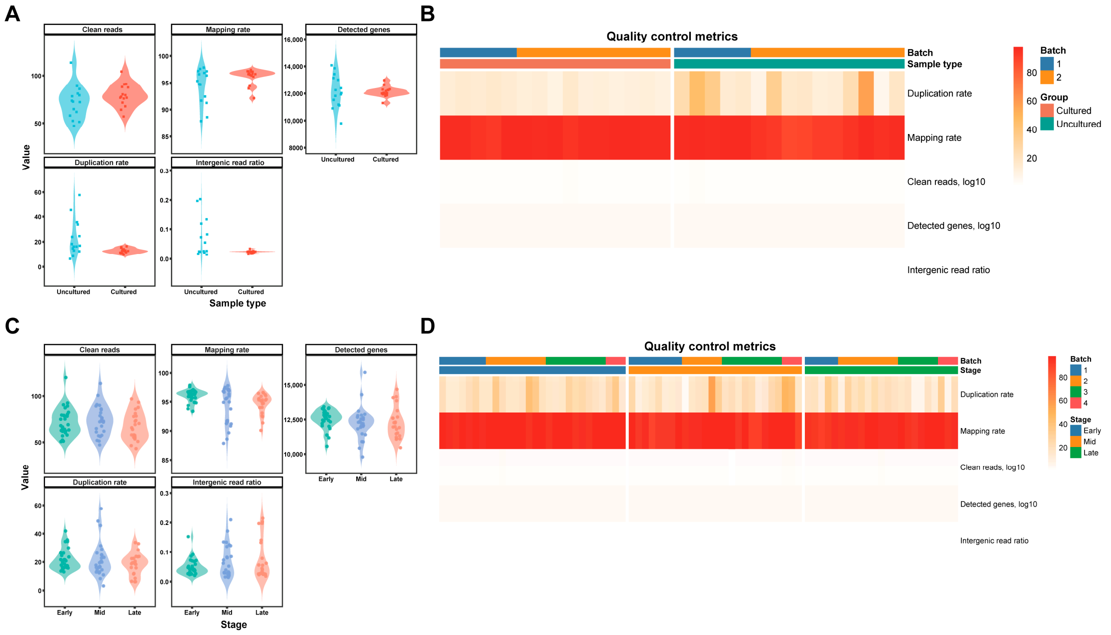

Figure S1. Sequencing quality control of amniocyte RNA-seq samples. (A) Violin plots of clean reads, mapping rate, detected genes, and duplication rate in paired uncultured and cultured amniocyte samples. **(B)** Heatmap summarizing the same quality control metrics in paired samples, with sequencing batch and sample type shown as annotations. Several uncultured samples had higher duplication rates, whereas clean reads, mapping rate, and detected gene numbers were generally comparable between the two sample types. **(C)** Violin plots of quality control metrics in uncultured amniocyte samples grouped by gestational stage: Early, Mid, and Late. **(D)** Heatmap of quality control metrics in uncultured amniocyte samples across gestational stages, with sequencing batch and stage shown as annotations. Overall QC profiles were comparable among the three stage groups, and sequencing batches were evenly distributed across stages.

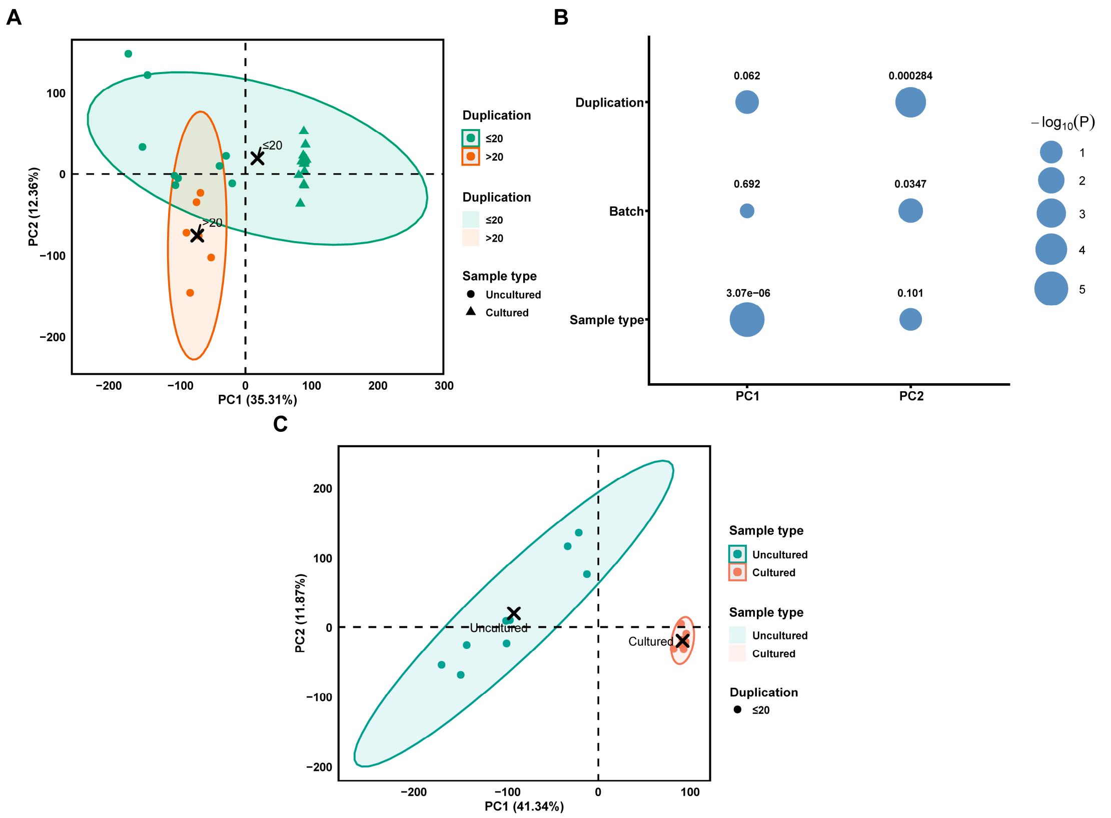

Figure S2. Sensitivity analysis for duplication rate in paired uncultured and cultured amniocyte samples. (A) PCA plot colored by duplication-rate group. Samples with different duplication rates showed a modest pattern along PC2. **(B)** Bubble plot of Kruskal–Wallis test results for associations between PC scores and duplication rate, sequencing batch, and sample type. PC1 was associated only with sample type ($P < 0.001$), whereas PC2 was associated with duplication rate and sequencing batch ($P < 0.05$), but not with sample type. **(C)** PCA after excluding samples with duplication rates >20%. Cultured and uncultured amniocytes remained separated, indicating that the sample type–related transcriptomic difference was not mainly driven by high-duplication samples.

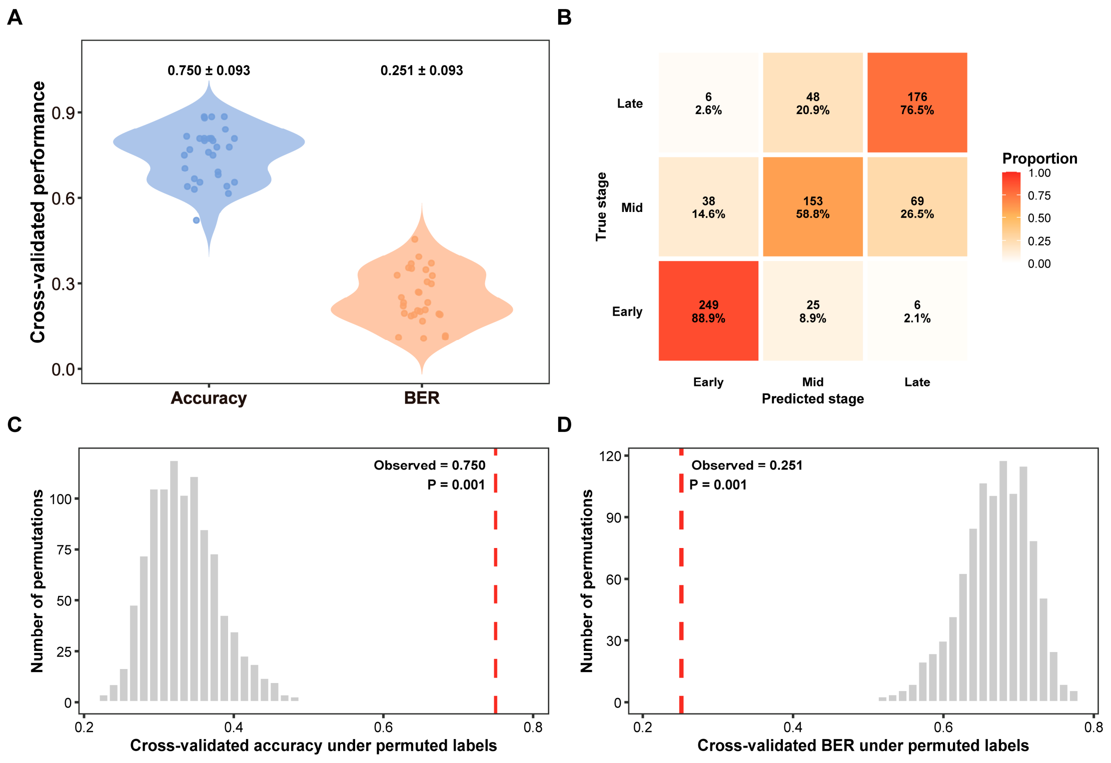

Figure S3. Assessment of fetal sex effects on uncultured amniocyte transcriptomic profiles. PCA of uncultured amniocyte expression profiles colored by fetal sex, showing no apparent sex-specific clustering pattern across the fetal sex.

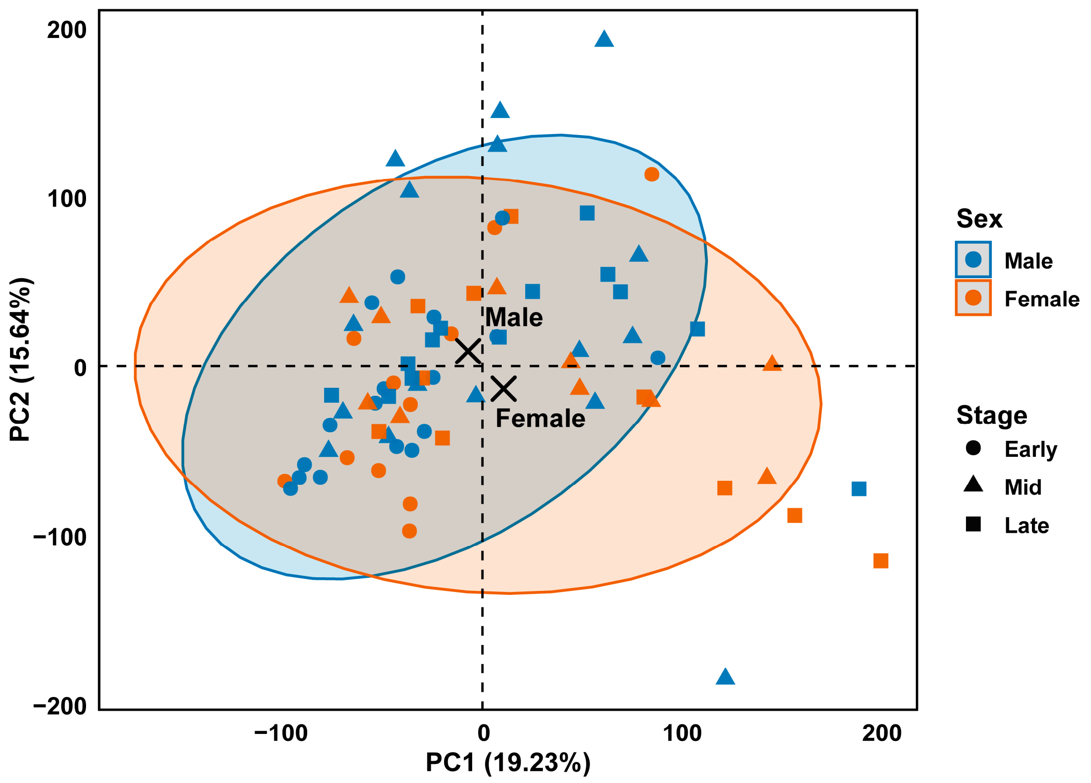

Figure S4. Batch-correction sensitivity analysis of uncultured amniocyte transcriptomic profiles. (A) PCA before batch adjustment, colored by sequencing batch. **(B)** PCA after batch adjustment, colored by sequencing batch. **(C)** PCA after batch adjustment, colored by gestational-stage group. **(D)** Kruskal–Wallis test results before and after batch adjustment. Gestational stage remained associated with PC1 after batch adjustment (P = 0.000226), whereas sequencing batch was not associated with PC1 or PC2. Duplication rate was associated mainly with PC2, indicating an effect on a secondary component of variation.

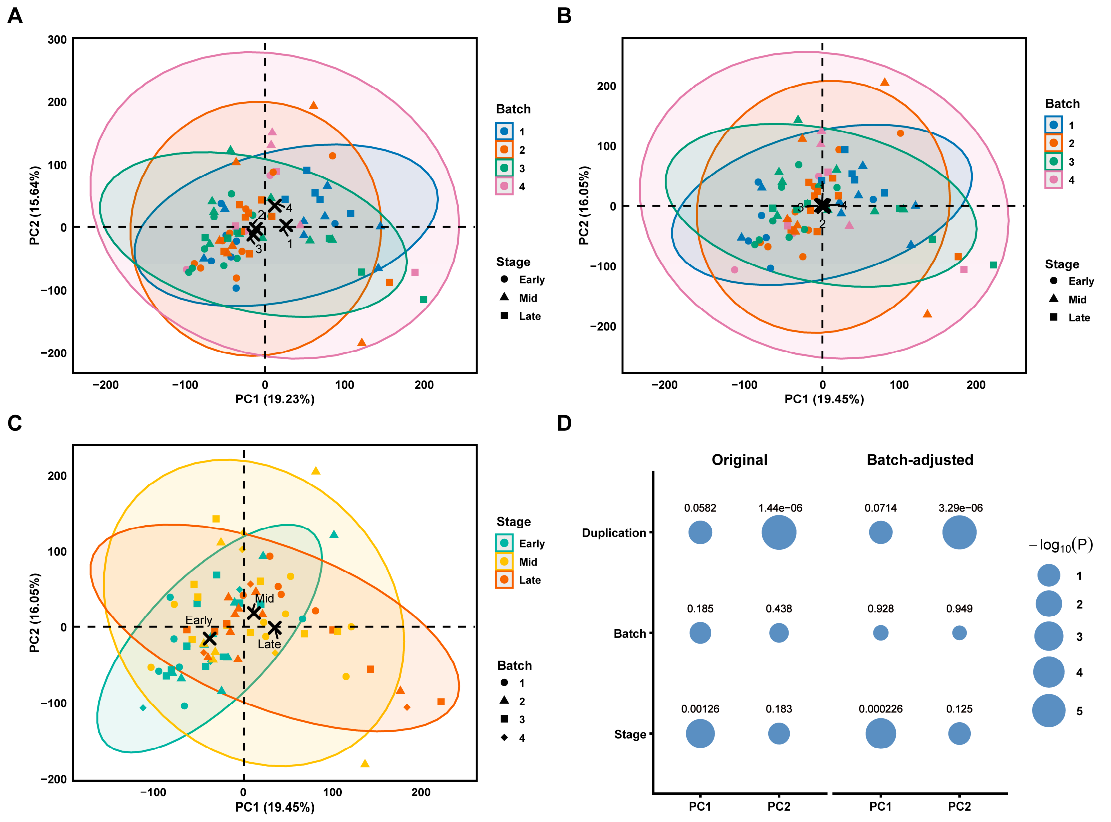

Figure S5. Cross-validation performance of the PLS-DA model for gestational stage classification. (A) Violin plots of cross-validated accuracy and balanced error rate (BER), with mean ± standard deviation indicated for each metric. **(B)** Cross-validated confusion matrix showing the classification performance of the PLS-DA model across the Early-, Mid-, and Late-stage groups. Early- and Late-stage samples were less frequently misclassified, whereas Mid-stage samples were more often assigned to adjacent gestational stages, consistent with a gradual transcriptomic transition across gestation. **(C)** Permutation test of cross-validated accuracy. The red dashed line indicates the observed accuracy of 0.750, which was higher than expected under permuted labels (P = 0.001). **(D)** Permutation test of cross-validated BER. The red dashed line indicates the observed BER of 0.251, which was lower than expected under permuted labels (P = 0.001).
