# Supplementary figures and images for: Uncultured Amniocytes Enable Rapid and Clinically Informative Prenatal RNA Sequencing for Genetic Diagnosis
Source: Int J Mol Sci. 2026 Jul 21;27(14):6465. doi: 10.3390/ijms27146465 (PMC13411854; doi:10.3390/ijms27146465)

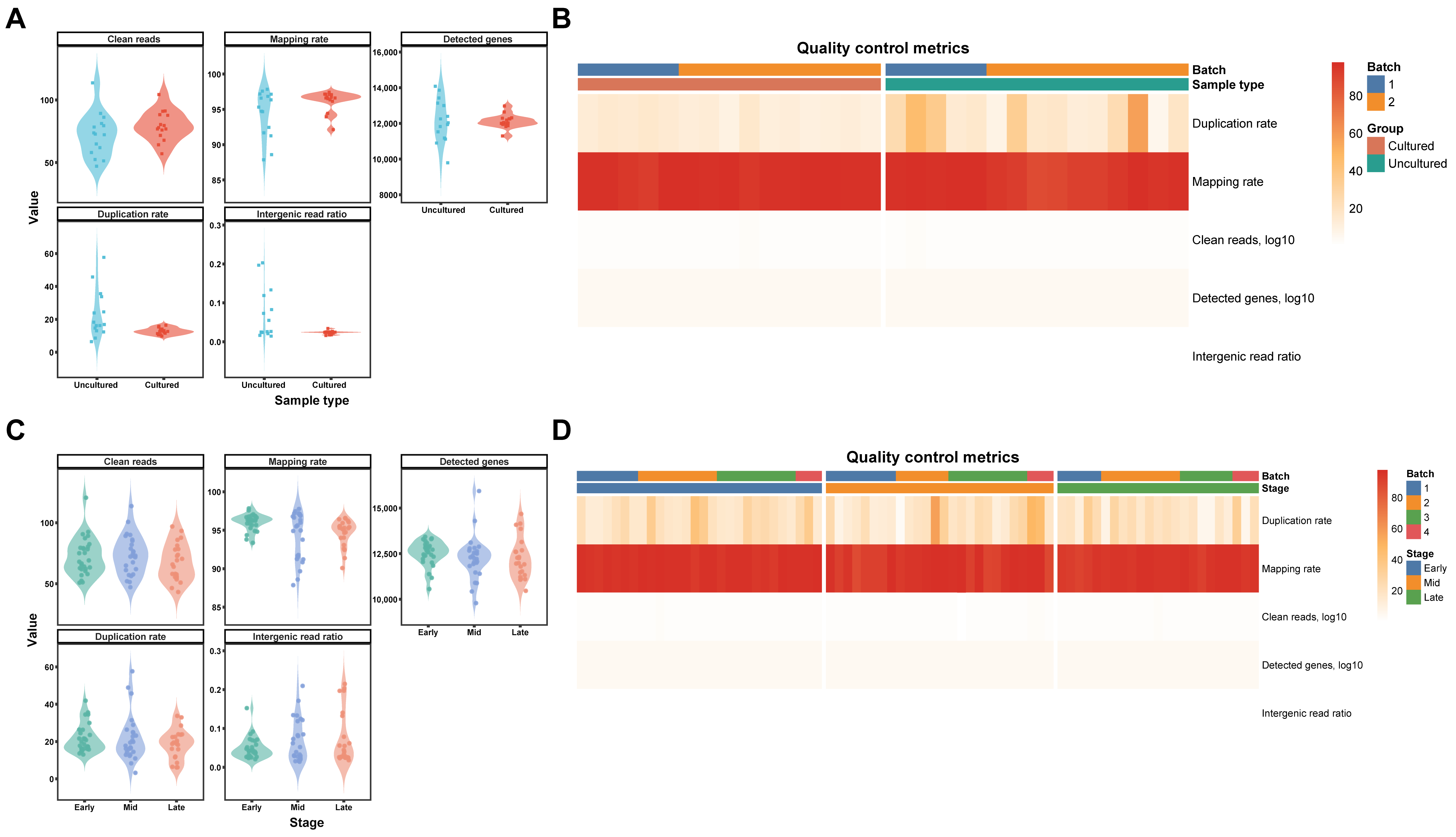

Supplement: Supplementary file 1 [file ijms-27-06465-s001.zip › Figure S1.tif]

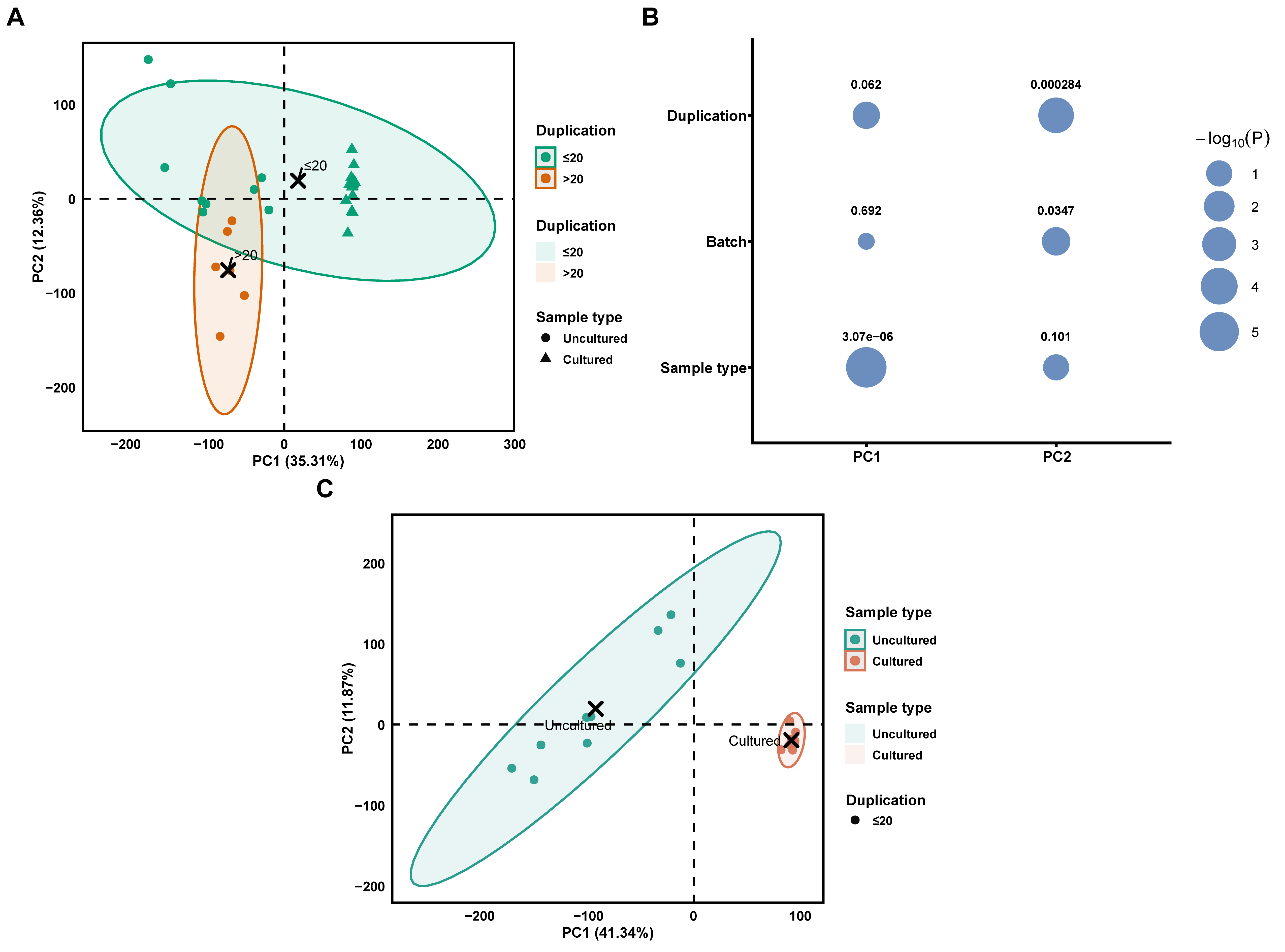

Supplement: Supplementary file 1 [file ijms-27-06465-s001.zip › Figure S2.tif]

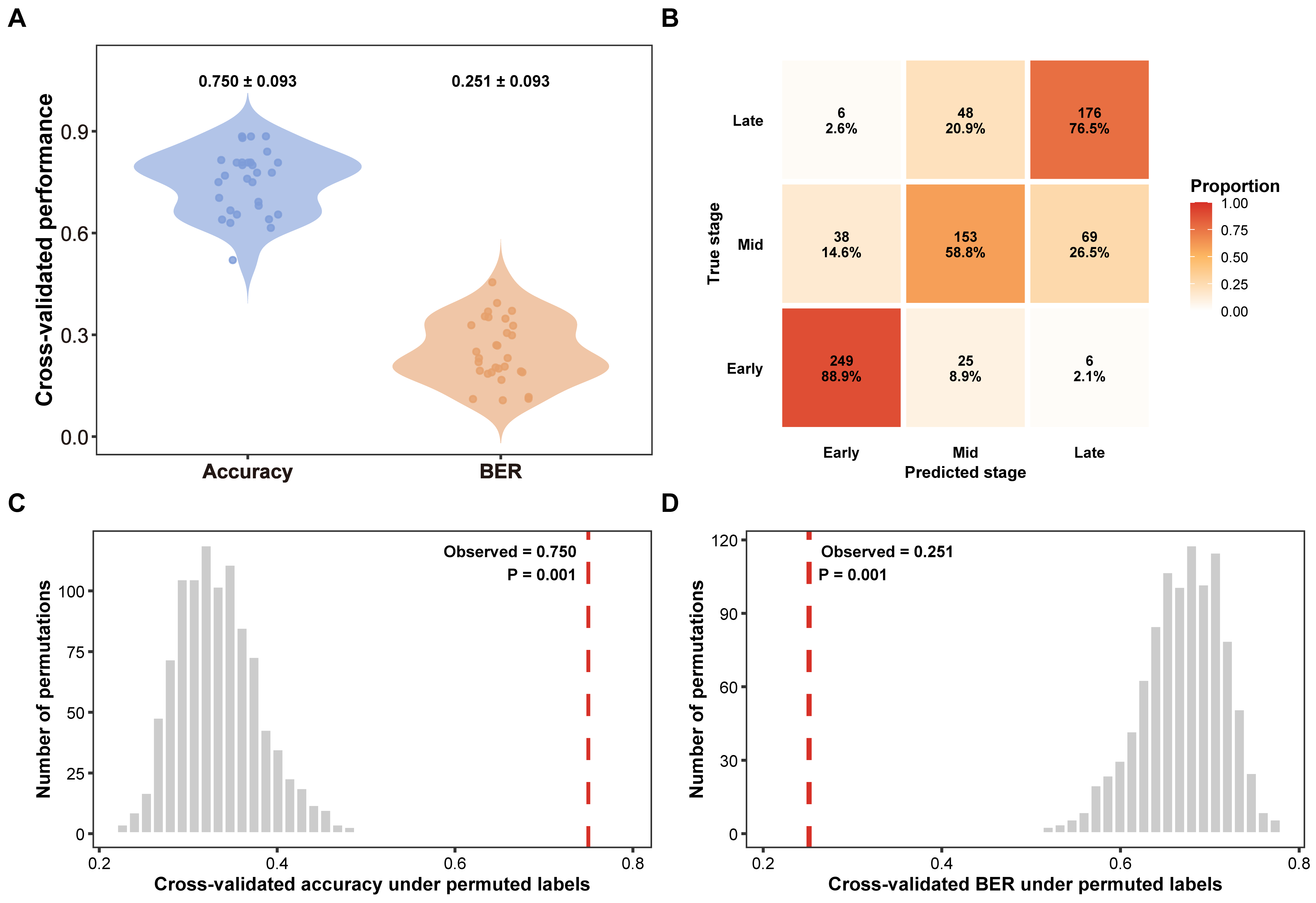

Supplement: Supplementary file 1 [file ijms-27-06465-s001.zip › Figure S3.tif]

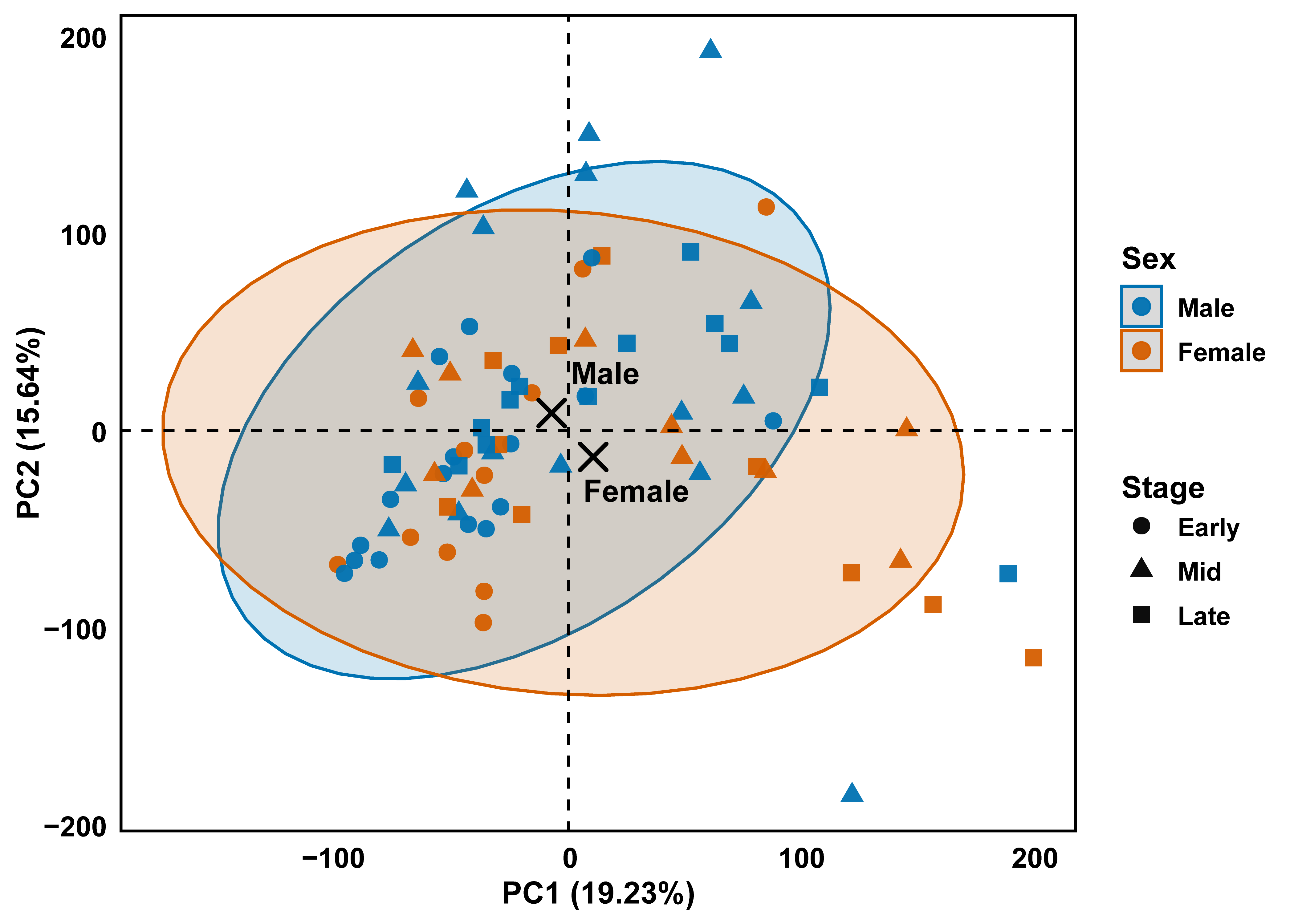

Supplement: Supplementary file 1 [file ijms-27-06465-s001.zip › Figure S4.tif]

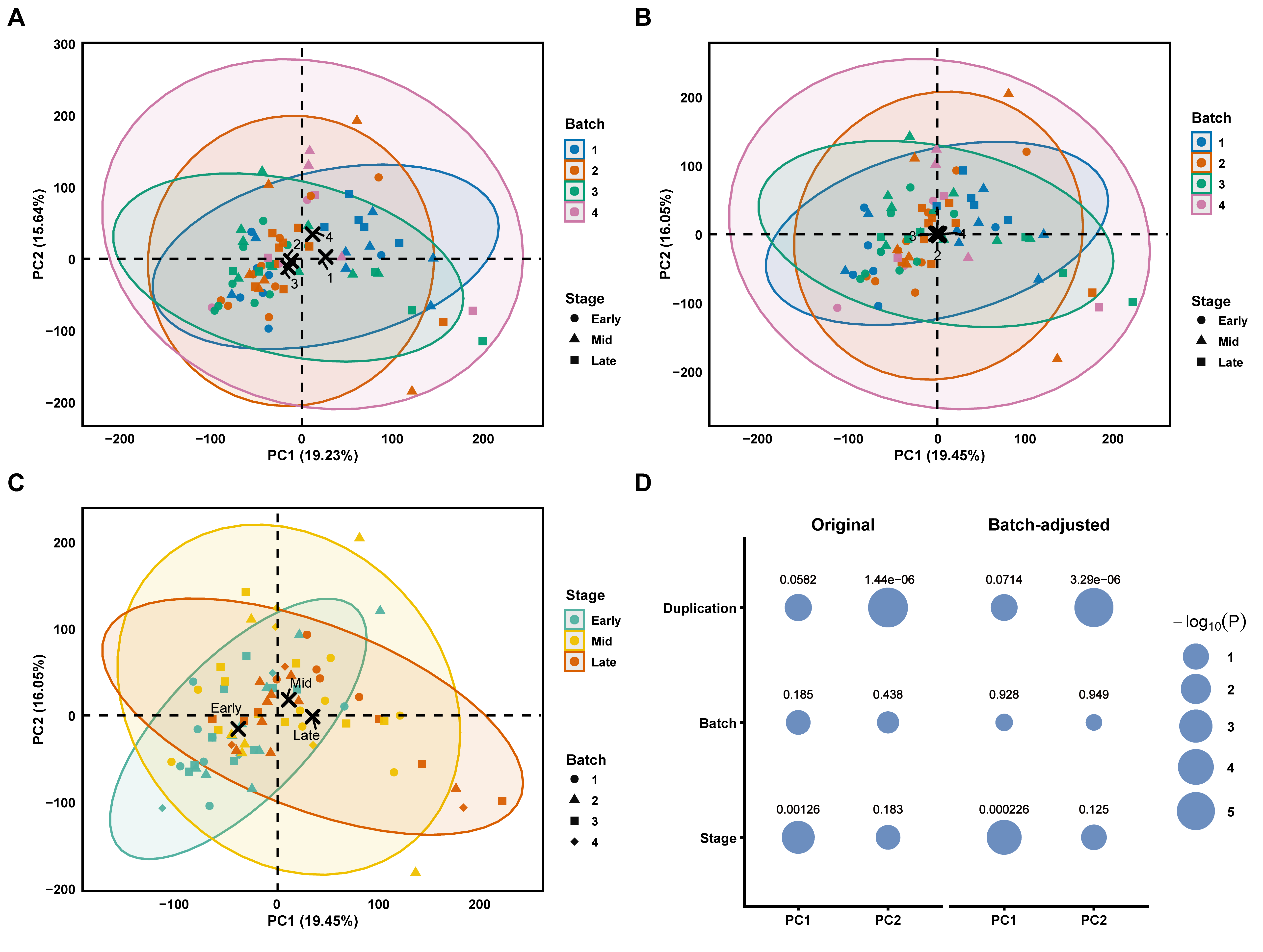

Supplement: Supplementary file 1 [file ijms-27-06465-s001.zip › Figure S5.tif]
